# Diffuse Glioneuronal Tumor With Oligodendroglioma-Like Features and Nuclear Clusters Relapse in a Seven-Year-Old Boy: An Unusual Case Exhibiting Near-Tetraploidy and Chromosome 14 Diploidy

**DOI:** 10.7759/cureus.73157

**Published:** 2024-11-06

**Authors:** Francis M Torres, Thomas Denapoli, Ping-Sun Chen, Carrie Mohila, Kevin E Fisher, Kenneth Aldape, Mark R Lee, Timothy C Griffin

**Affiliations:** 1 Hematology and Oncology Clinical Research, CHRISTUS Children's, San Antonio, USA; 2 Pediatric Pathology and Cytopathology, CHRISTUS Children's, San Antonio, USA; 3 Neuroradiology, CHRISTUS Children's, San Antonio, USA; 4 Neuropathology, Texas Children's Hospital, Baylor College of Medicine, Houston, USA; 5 Molecular Genetic Pathology, Texas Children's Hospital, Baylor College of Medicine, Houston, USA; 6 Molecular Neuropathology, National Institutes of Health, Bethesda, USA; 7 Neurosurgery, CHRISTUS Children's, Baylor College of Medicine, San Antonio, USA; 8 Hematology and Oncology, CHRISTUS Children's, Baylor College of Medicine, San Antonio, USA

**Keywords:** brain tumor, dna methylation classification, glioneuronal tumor, monosomy 14, pediatric, tetraploid

## Abstract

Diffuse glioneuronal tumor with oligodendroglioma-like features and nuclear clusters (DGONC) is a rare brain tumor of the central nervous system (CNS). Although only a few cases of DGONC have been reported following the initial description of the tumor, they have a distinct DNA methylation pattern and share a recurrent chromosomal finding of monosomy 14. We encountered a seven-year-old boy who presented with seizures and was found to have a left frontal and suprasellar mass. The tumor was grossly totally resected; histopathologic evaluation showed a cellular glioneuronal tumor with brisk mitotic activity. A near-tetraploid chromosome complement was detected, with associated diploidy of chromosome 14. Methylation pattern analysis revealed findings consistent with DGONC. Initially, we observed the patient without additional therapy. Due to the patient's non-metastatic relapse, resection of the relapse tumor consistent with DGONC and adjuvant radiotherapy were then initiated. The natural history and optimal post-surgical adjuvant therapy are unknown. This case adds to the limited number of DGONC cases previously reported, with a unique pattern of chromosomal abnormalities.

## Introduction

Making an accurate pathological diagnosis of central nervous system (CNS) tumors can be challenging, but it is vital to provide clinicians with data that informs optimal patient management. Beyond histopathology and molecular evaluation of tumor deoxyribonucleic acid (DNA) for specific genetic changes, more recently, the delineation of tumor-specific DNA methylation signatures has permitted the classification of tumors into molecularly distinct subgroups. One such example is the diffuse glioneuronal tumor with oligodendroglioma-like features and nuclear clusters (DGONC), an entity first delineated in 31 patients in a retrospective molecular analysis performed by Deng et al. and a multi-national collaborative in 2020 [1]. This DNA methylation-defined tumor was found to have certain recurring neuropathological traits and the strikingly consistent finding of monosomy 14, with the vast majority of the patients being in the pediatric age group [1]. Previous historical diagnoses in that initial cohort included various entities of variable grade. Their compelling observations led to the inclusion of the DGONC into the 2021 revision of the WHO classification of CNS tumors as a provisional entity [2]. Subsequently, with the histopathological features and methylation profiling, four more cases of DGONC appeared in the literature [3,4].

We recently encountered a seven-year-old boy who presented with seizures and was found to have a suprasellar/left inferior frontal brain tumor. As with many cases of DGONC, imaging characteristics and initial traditional pathologic evaluation were somewhat confusing. DNA analysis revealed an unusual near-tetraploidy, albeit with only two copies of chromosome 14. DNA methylation pattern analysis helped establish the diagnosis of DGONC. Based on our search, only a few cases were reported, adding to the limited number of DGONC cases previously reported with a unique pattern of chromosomal abnormalities.

This article was previously presented as a poster at the 2024 American Association of Neuropathologists (AANP) Centennial meeting on June 6-9, 2024.

## Case presentation

**Figure 1:**
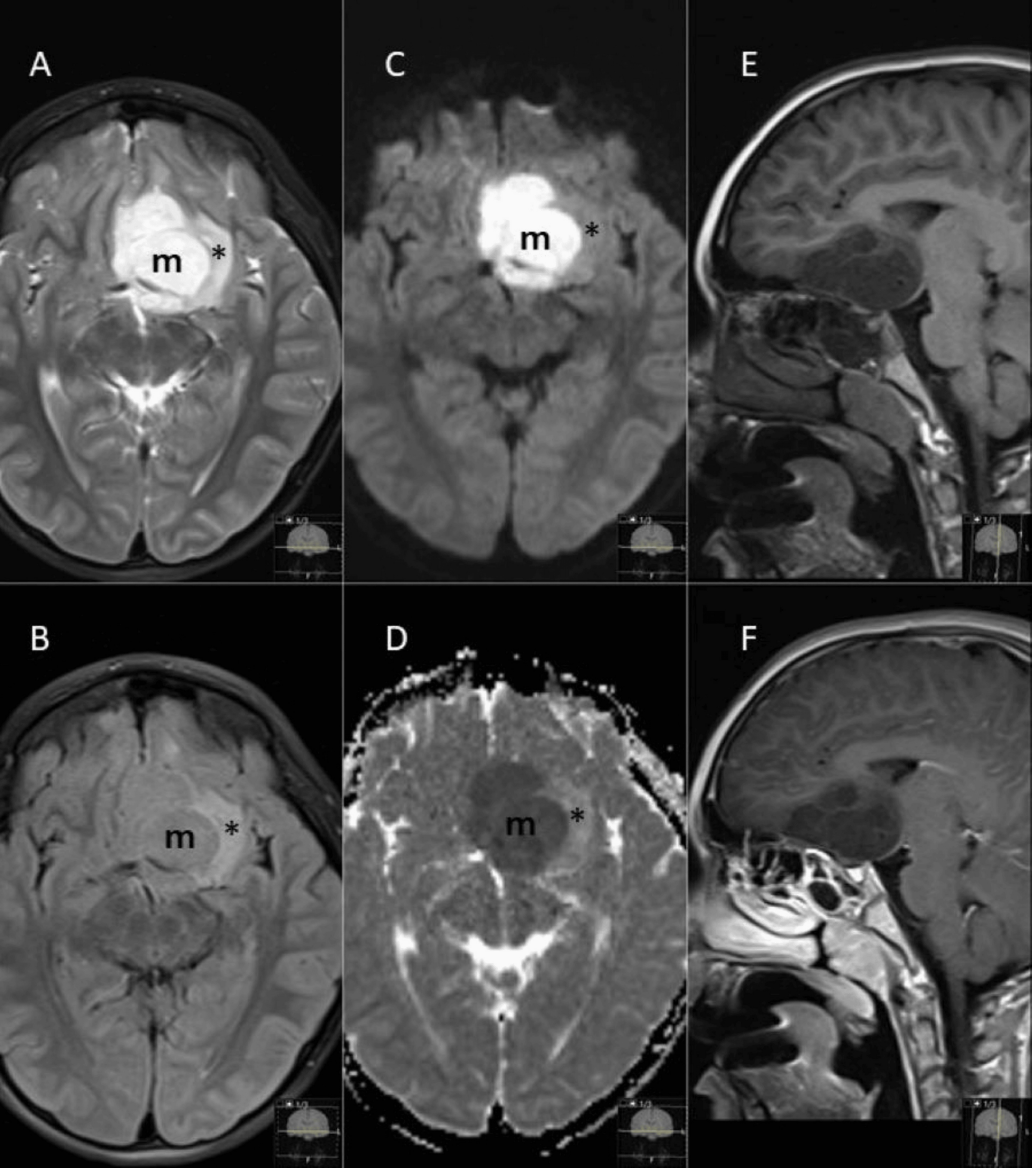
MRI of the brain revealed a 35 x 41 x 38-mm intra-axial mass (m) at the left inferior frontal lobe situated atop the planum sphenoidale, encasing the left anterior cerebral artery (ACA) and deviating the optic chiasm and left optic tract. The mass was hyperintense on T2 (A) and isointense on T2/FLAIR (B). Diffusion-weighted imaging (DWI) (C) showed intense proton diffusion restriction with a low apparent diffusion coefficient (ADC) (D). The mass was hypointense on T1 (E) and showed no enhancement after gadolinium on post-contrast T1 (F). Minimal perilesional vasogenic edema can be seen at the periphery (*). T2/FLAIR: T2-weighted fluid-attenuated inversion recovery.

A seven-year-old boy presented with a new onset of tonic-clonic seizures. A month before the onset of the new seizure, the patient had one episode of febrile seizure and noticed a change in his mood. He is more aggressive at school. Electroencephalogram revealed near-constant spikes and spike-and-wave complexes over the left frontotemporal region. The findings were concerning for an underlying structural lesion. Magnetic resonance imaging (MRI) of the brain revealed an intra-axial mass at the left inferior frontal lobe atop the planum sphenoidale measuring 35 x 41 x 38 mm. The mass encased the left anterior cerebral artery, deviating the optic chiasm and left optic tract. The mass was T1 hypointense and T2 hyperintense relative to gray matter and isointense on the T2-weighted fluid-attenuated inversion recovery (T2/FLAIR) sequence. Diffusion-weighted imaging (DWI) showed the entire mass demonstrating intense proton diffusion restriction with a low apparent diffusion coefficient (ADC). No enhancement was noted after gadolinium contrast. No calcification was noted on CT or MRI. There was minimal perilesional vasogenic edema. The imaging characteristics of the MRI and the magnetic resonance spectroscopy findings suggested that the lesion was possibly an epidermoid cyst (Figure 1).

**Figure 2:**
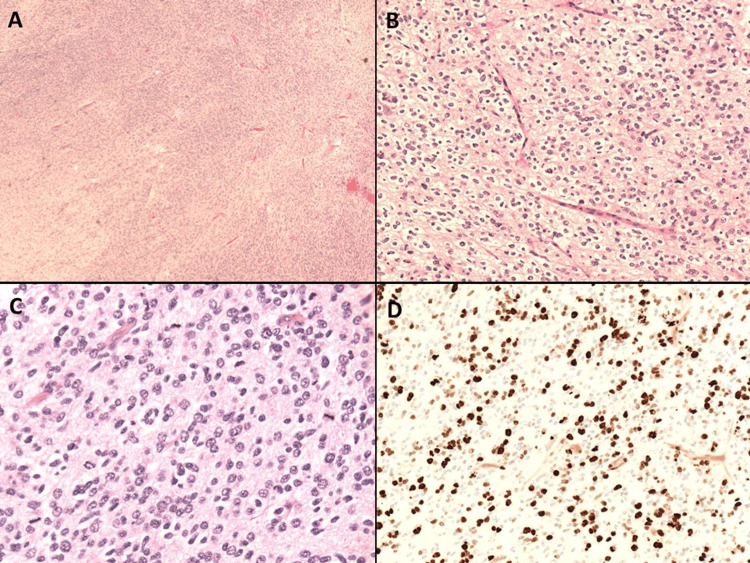
Microscopic examination revealed a hypercellular infiltrating tumor (A) composed of cells with round, mildly pleomorphic, hyperchromatic nuclei with focal prominent pericellular clearing imparting an oligodendroglioma-like appearance (B). Rare nuclear clusters were seen, and rare perivascular pseudorosettes were identified. Mitotic activity was brisk with at least 27 mitotic figures per 10 high-power fields (C). The Ki67 proliferative index was elevated to approximately 40% in the most proliferative areas (D).

An ophthalmology evaluation showed that visual fields were grossly normal. Three months after the new onset of seizure, the patient underwent a craniotomy, and a gross total resection of the tumor was achieved. Pathological evaluation of the tumor using histopathological features (Figure 2) showed diffuse immunoreactivity for oligodendrocyte transcription factor 2 (OLIG-2), weak to moderate immunoreactivity for neuronal nuclear protein (Neu-N), and focal immunoreactivity for CD56. Immunostaining with glial fibrillary acidic protein (GFAP) was negative.

The tumor tissue was sent for methylation-based tumor profiling (Table 1), and a consensus methylation profile supports the diagnosis of DGONC. Post-operatively, the patient recovered uneventfully and was seizure-free but continued to have hyperactive and impulsive behavior, which improved after modification of his anticonvulsant medications. A follow-up MRI scan performed 3.5 months post-operation revealed resolution of post-operative changes and no evidence of tumor recurrence.

**Table 1:** The DNA methylation profile indicates a consensus match to diffuse glioneuronal tumor with oligodendroglioma-like features and nuclear clusters.

DNA methylation-based tumor classification	
Specimen source	Brain, suprasellar tumor
Histological classification	High-grade neuroepithelial tumor
Methylation class name	Diffuse glioneuronal tumor with oligodendrioglioma-like features and nuclear clusters (DGONC)
Methylation class confidence score	High confidence
Methylation class description	Diffuse glioneuronal tumor with oligodendrioglioma-like features and nuclear clusters (DGONC) comprises tumors morphologically characterized by diffuse growth, frequently clear cell histology, and formation of clusters of nuclei. These tumors virtually all harbor monosomy of chromosome 14

**Figure 3:**
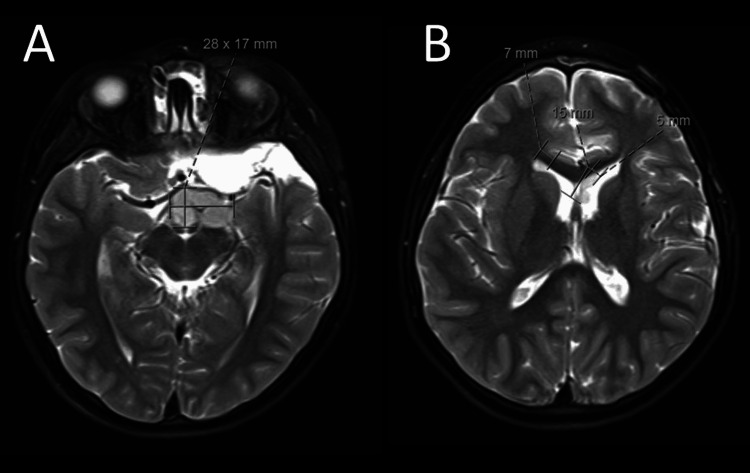
One year post-intervention, brain MRI revealed local tumor recurrence at the suprasellar cistern extending to the interpeduncular cistern and the anterior aspect of the third ventricle. The recurrent mass demonstrates a lobulated irregular margin, but the bulk of the mass is estimated at 27.8 mm x 7.4 mm axially and 12.7 cm craniocaudally at the level of the suprasellar cistern. A portion of the suprasellar component demonstrates diffusion restriction as the initial mass (A). There are nodular foci with similar imaging characteristics with a 7.3-mm nodular focus at the right anterior horn and 15 mm x 4.8 mm nodular foci at the left anterior lateral ventricle abutting the septum pellucidum (B).

An MRI scan almost a year post-operation showed a recurrence both in the original tumor bed and tumor seeding in the lateral ventricles (Figure 3). A resection of the original tumor bed was performed. Metastatic workup was negative. The pathology report was again consistent with DGONC. Adjuvant radiotherapy was administered with a resolution of the ventricular tumor, and the patient is again undergoing surveillance for recurrence. 

## Discussion

The recent discovery of DNA methylation testing on CNS tumors helps pathologists properly delineate the diagnosis of DGONC. DGONC is a newly recognized rare tumor of the CNS included as a provisional diagnosis in the 2021 5th WHO Classification of Tumors of the CNS [2]. DGONC was identified as a specific entity by profiling CNS tumors using DNA methylation-based classification, based on the work of Capper et al. [5] with the subsequent inclusion of chromosomal copy number data into the model [6]. Extending this methodology to a multi-center-derived Heidelberg cohort of >25,000 specimens, Deng et al. [1] delineated 31 CNS tumor cases with a novel DNA methylation-based profile, constituting the first DGONC cases to be characterized. Subsequently, four additional cases of DGONC have been reported. Pickles et al. [3] reviewed a cohort of 123 cases with monosomy 14 and previously indeterminate CNS tumor final diagnoses. Three of those cases, previously diagnosed as malignant glioneuronal tumors or high-grade neuroepithelial tumors, were identified with a methylation pattern matching that of DGONC. More recently, an additional case report with clinical, pathologic, chromosomal, and molecular characteristics has been published [4].

In cases reported thus far in the literature, DGONC mainly affects children (mean age of nine in the Deng et al. series) but has been seen in older individuals [1]. There appears to be no gender predilection. It mainly affects the cerebral hemispheres in the cortical or subcortical area, often involving the temporal lobe, and typically presents without neuraxis dissemination [1-3].

The name DGONC acronym is derived from the histological features, including (D) diffuse growth, (G) co-occurrence of glial and neuronal differentiation, (O) features of oligodendroglioma-like perinuclear halos, and (N, C) nuclear clusters resembling “pennies on a plate” [7]. Other variable histopathological features include inflammatory cells, neutrophil-like islands in the perivascular region, focal lymphocytic infiltration, macrophages, giant cells, perivascular pseudorosettes, or calcification. On immunohistochemistry, DGONC typically exhibits diffuse positivity for OLIG-2 and synaptophysin and focal positivity for Neu-N and microtubule-associated protein 2 (MAP2), while being negative for GFAP [8].

Despite the trends in immunohistochemistry noted above, retrospective reviews of previously assigned histopathologic diagnoses in cases subsequently identified as DGONC highlight the challenges in establishing the diagnosis by routine methods. As in our case, a high mitotic rate and proliferative index are often seen, which explains why many cases of DGONC were previously classified as high-grade tumors. DGONC appears to be an entity that is uniquely identifiable by its methylation pattern, presence of monosomy 14, and concomitant absence of the genetic alterations found in other glioneuronal tumors, including alterations of V-Raf murine sarcoma viral oncogene homolog B (BRAF), fibroblast growth factor receptor (FGFR), and isocitrate dehydrogenase (IDH) genes.

**Figure 4:**
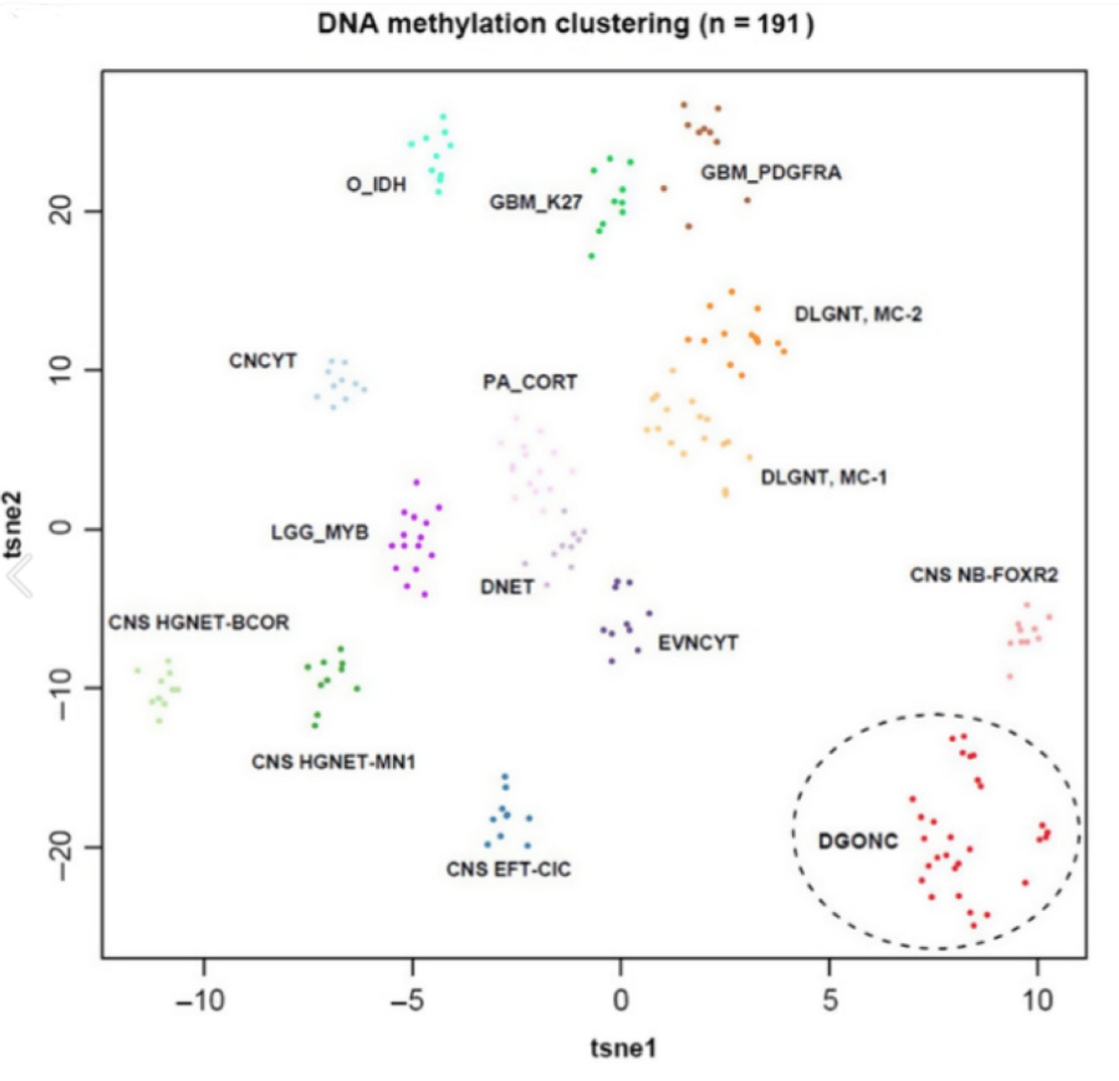
DNA methylation profiling of DGONC from the Deng et al. paper. DGONC: diffuse glioneuronal tumor with oligodendroglioma-like features and nuclear clusters. Reuse of this image with permission from WILEY Company, license # 5627160729091. Source: reference [1].

**Figure 5:**
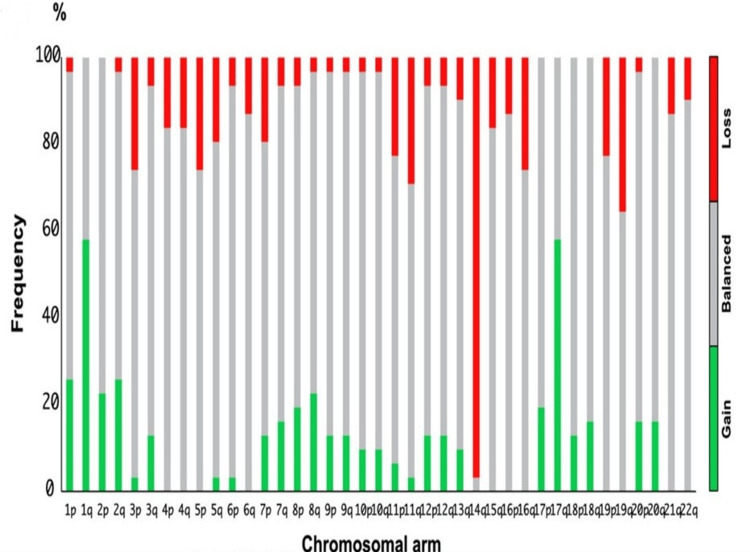
Summary of copy number profiles of cases derived from DNA methylation chromosomal gains and losses of cases, 97% of cases with monosomy 14. Reuse of this image with permission from WILEY Company, license # 5627160729091. Source: reference [1].

A striking characteristic finding in DGONC is monosomy 14 (Figures 4, 5) [1], seen in all but one of the cases reported by Deng et al. and in all four cases subsequently described. Our case appears to be unique in finding a near-tetraploid chromosome complement, with the diploid presence of chromosome 14. This suggests that the loss of chromosome 14 preceded a subsequent duplication of an aneuploid clone. The significance of this observation, if any, is unknown. Our case also exhibited other chromosomal alterations often seen in DGONC, including gains of chromosome arms 1q and 17q and a loss of chromosome arm 19q [1].

The optimal post-operative management of DGONC cases remains undefined. While Deng et al.’s original paper indicates that the overall outcomes were “relatively favorable,” the prognosis must be considered guarded. Of the 12 of 31 patients with clinical follow-up, three had evidence of progression, and two died; no details of adjuvant treatment were provided. Surgical removal seems to be of primary importance, although in at least one instance, residual tumor following partial resection has evidenced no progression over 17 months [4]. The three cases reported by Pickles et al. achieved complete gross tumor resection, with all remaining relapse-free after receiving craniospinal irradiation and unspecified chemotherapy [3]. Additional outcome data will be required to determine the best adjunctive approach. As our patient had a complete resection and given his pre-existing neurobehavioral issues, in light of the case mentioned above [4], we chose to initially observe him closely with no additional therapy. However, due to his non-metastatic relapse, he has undergone re-resection with adjuvant radiotherapy, with an initial encouraging response.

The limitation of the study is that since DGONC is a new provisional diagnosis of CNS tumor, and due to the rarity of cases, the additional therapy after surgery is still unknown.

## Conclusions

This report of a rare case of DGONC is a unique subset of glioneuronal tumors that is challenging to diagnose using traditional methods and most accurately diagnosed using DNA methylation methodology, including chromosome copy number analysis and select DNA genomic testing to exclude other similar CNS tumors. The significance of the consistent finding of monosomy 14 in DGONC is unknown. Much more information is required to delineate what, if any, adjuvant therapy is necessary beyond surgical resection.
